# Prediabetes Prevalence by Adverse Social Determinants of Health in Adolescents

**DOI:** 10.1001/jamanetworkopen.2024.16088

**Published:** 2024-06-11

**Authors:** Caleb Harrison, Vaishnavi Peyyety, Adriana Rodriguez Gonzalez, Rutha Chivate, Xu Qin, Margaret F. Zupa, Maya I. Ragavan, Mary Ellen Vajravelu

**Affiliations:** 1 Center for Pediatric Research in Obesity and Metabolism, UPMC Children’s Hospital of Pittsburgh, Pittsburgh, Pennsylvania; 2 Division of Pediatric Endocrinology, Diabetes, and Metabolism, University of Pittsburgh School of Medicine, Pittsburgh, Pennsylvania; 3 Washington & Jefferson College, Washington, Pennsylvania; 4 University of Pittsburgh School of Medicine, Pittsburgh, Pennsylvania; 5 Department of Health and Human Development at the School of Education, University of Pittsburgh, Pittsburgh, Pennsylvania; 6 Division of Endocrinology and Metabolism, University of Pittsburgh School of Medicine, Pittsburgh, Pennsylvania; 7 Division of General Academic Pediatrics, University of Pittsburgh Medical Center Children’s Hospital of Pittsburgh, Pittsburgh, Pennsylvania

## Abstract

**Importance:**

Several clinical practice guidelines advise race- and ethnicity-based screening for youth-onset type 2 diabetes (T2D) due to a higher prevalence among American Indian and Alaska Native, Asian, Black, and Hispanic youths compared with White youths. However, rather than a biological risk, this disparity likely reflects the inequitable distribution of adverse social determinants of health (SDOH), a product of interpersonal and structural racism.

**Objective:**

To evaluate prediabetes prevalence by presence or absence of adverse SDOH in adolescents eligible for T2D screening based on weight status.

**Design, Setting, and Participants:**

This cross-sectional study and analysis used data from the 2011 to 2018 cycles of the National Health and Nutrition Examination Survey. Data were analyzed from June 1, 2023, to April 5, 2024. Participants included youths aged 12 to 18 years with body mass index (BMI) at or above the 85th percentile without known diabetes.

**Main Outcomes and Measures:**

The main outcome consisted of an elevated hemoglobin A_1c_ (HbA_1c_) level greater than or equal to 5.7% (prediabetes or undiagnosed presumed T2D). Independent variables included race, ethnicity, and adverse SDOH (food insecurity, nonprivate health insurance, and household income <130% of federal poverty level). Survey-weighted logistic regression was used to adjust for confounders of age, sex, and BMI *z* score and to determine adjusted marginal prediabetes prevalence by race, ethnicity, and adverse SDOH.

**Results:**

The sample included 1563 individuals representing 10 178 400 US youths aged 12 to 18 years (mean age, 15.5 [95% CI, 15.3-15.6] years; 50.5% [95% CI, 47.1%-53.9%] female; Asian, 3.0% [95% CI, 2.2%-3.9%]; Black, 14.9% [95% CI, 11.6%-19.1%]; Mexican American, 18.8% [95% CI, 15.4%-22.9%]; Other Hispanic, 8.1% [95% CI, 6.5%-10.1%]; White, 49.1% [95% CI, 43.2%-55.0%]; and >1 or other race, 6.1% [95% CI, 4.6%-8.0%]). Food insecurity (4.1% [95% CI, 0.7%-7.5%]), public insurance (5.3% [95% CI, 1.6%-9.1%]), and low income (5.7% [95% CI, 3.0%-8.3%]) were each independently associated with higher prediabetes prevalence after adjustment for race, ethnicity, and BMI *z* score. While Asian, Black, and Hispanic youths had higher prediabetes prevalence overall, increasing number of adverse SDOH was associated with higher prevalence among White youths (8.3% [95% CI, 4.9%-11.8%] for 3 vs 0.6% [95% CI, −0.7% to 2.0%] for 0 adverse SDOH).

**Conclusions and Relevance:**

Adverse SDOH were associated with higher prediabetes prevalence, across and within racial and ethnic categories. Consideration of adverse SDOH may offer a more actionable alternative to race- and ethnicity-based screening to evaluate T2D risk in youth.

## Key Points

**Question:**

How does youth-onset prediabetes prevalence differ by exposure to adverse social determinants of health (SDOH), independent of race and ethnicity?

**Findings:**

In this cross-sectional study including a nationally representative sample of 1563 adolescents aged 12 to 18 years with obesity, the SDOH categories of food insecurity, lack of private health insurance, and lower household income were associated with higher prediabetes prevalence, independent of race and ethnicity.

**Meaning:**

These findings suggest that adverse SDOH should be recognized in clinical settings and used to guide efforts to reduce risk of youth-onset type 2 diabetes.

## Teaser

This cross-sectional study evaluates differences in prediabetes prevalence by intersectional social determinants of health (SDOH) and race or ethnicity categories in a nationally representative sample of US adolescents eligible for type 2 diabetes screening based on age and body mass index–based overweight or obesity.

## Introduction

Type 2 diabetes (T2D) is a disease strongly influenced by poverty and structural racism.^1,2^ For the past 2 decades, a rapidly rising incidence in youth-onset T2D, particularly among American Indian or Alaska Native, Asian, Black, and Hispanic youths,^3^ has highlighted the urgent need to prevent the onset of this severe disease, which leads to at least 1 microvascular complication by the third to fourth decades of life in 80% of individuals.^4^ One potential strategy to stem the tide of youth-onset T2D is to limit the development of youth-onset prediabetes, an intermediate glycemic state that is also associated with cardiometabolic comorbidities and obesity.^5,6^ Youth-onset prediabetes has more than doubled in prevalence in the past 2 decades and is now present in 28% of US adolescents overall and 40% of adolescents with obesity.^7^ Like youth-onset T2D, prediabetes is more common among racial and ethnic minority youths,^6^ and it may be more likely to progress to T2D in non-Hispanic Black youths than in Hispanic or non-Hispanic White youths.^8^

However, these epidemiological observations may be driven by social determinants of health (SDOH), which are conditions in which people grow, live, and work that are shaped by the distribution of money, power, and resources at global, national, and local levels.^2^ These conditions can be divided into 5 broad categories: educational access and quality, health care access and quality, neighborhood and built environments, economic stability, and social and community context.^2^ Exposure to adverse SDOH differs by the social, nonbiological constructs of race and ethnicity.^2,9^ Although previous studies have demonstrated associations between adult-onset T2D and adverse SDOH,^2,10^ the potential interactive roles of race, ethnicity, and SDOH in youth-onset prediabetes have not been evaluated.^11,12^ Racial and ethnic minority youths are more likely to experience adverse SDOH related to income inequality,^13^ so understanding the intersection between race and ethnicity and SDOH is critical to reduce T2D risk. This is particularly true for youths with overweight or obesity, who are at the highest risk for prediabetes and T2D.^14^

To address this gap, we evaluated differences in prediabetes prevalence by intersectional SDOH and race or ethnicity categories in a nationally representative sample of American adolescents who would be eligible for T2D screening based on age and body mass index (BMI)–based classification of overweight or obesity.^5^ We used the Healthy People 2030 SDOH domains^15^ to guide our selection of measures available in the National Health and Nutrition Examination Survey (NHANES) from 2011 to 2018. We hypothesized that adverse SDOH (food insecurity, lack of private health insurance, and lower household income) would be associated with higher prediabetes prevalence, both within and across racial and ethnic groups, partially explaining the observed racial disparities in prevalence due to higher rates of adverse SDOH among minoritized populations.

## Methods

### Study Population

The study followed the Strengthening the Reporting of Observational Studies in Epidemiology (STROBE) reporting guideline. This cross-sectional analysis included youths aged 12 to 18 years with BMI at or above the 85th percentile for age and sex and with available hemoglobin A_1c_ (HbA_1c_) level measurements in 2-year survey cycle waves from 2011 to 2018 of NHANES. NHANES is a large program conducted by the National Center for Health Statistics that collects demographic, socioeconomic, and health-related surveys as well as physical measurements and clinical laboratory evaluations in a selected population that is designed to be representative of the US population after survey weighting. Administration of each NHANES questionnaire is based on participant age and sex, as relevant. Participants 16 years or older are interviewed directly, while an adult proxy (eg, parent or guardian) provided information on behalf of the survey participant for those younger than 16 years or who cannot answer questions themselves.

We began with data from the 2011-2012 cycle due to addition of a non-Hispanic Asian race and ethnicity category that began in that cycle, as Asian youth are at significantly increased risk of T2D.^3^ More recent data from the 2019-2020 cycle are not considered to be nationally representative due to limitations in data collection during the COVID-19 pandemic, so were not included in this analysis. We chose an age range of 12 to 18 years to be inclusive of a youth population that would be eligible for screening for T2D based on American Diabetes Association guidelines, as well as the availability of HbA_1c_ levels in the dataset (beginning at 12 years of age). Participants with known diabetes based on response to diabetes risk-related survey questions were excluded (eFigure in Supplement 1). All NHANES survey cycles were approved by the National Center for Health Statistics Research Ethics Review Board. Informed consent was obtained from parents or legal guardians or assent from youths 16 years or older prior to participation in NHANES. Survey question numbers and phrasing are listed in the eTable in Supplement 1.

### Independent Variables

#### Social Determinants of Health

We included 3 SDOH measures consistent with Healthy People 2030 domains of economic stability and health care access: food security, household income, and health insurance. These domains are also highly relevant to prediabetes management. Families with food insecurity are less likely to have access to nutrient-rich food to prevent prediabetes; those without private insurance may have more limited access to care; and those living in poverty may have both food insecurity and lack of private insurance, as well as fewer opportunities to engage in physical activity due to neighborhood-level barriers. We used the Food Security Questionnaire to determine the household food security level, defined by number of affirmative responses to the US Food Security Survey Module questions (0 indicates full security; 1-2, marginal; 3-7, low; and 8-18, very low); this was dichotomized to full vs not full (marginal, low, or very low) food security. We chose to include marginal with food insecurity based on evidence that marginal food security is associated with adverse child health outcomes^16^ as well as with poor glycemic control in adult NHANES participants.^17^ To assess health care access, we used the Health Insurance Questionnaire to determine whether each individual had public, private, other, or no insurance. This variable was analyzed as any private insurance, only public insurance (or other, including single-service plans), and no insurance. The family poverty-income ratio, reported on the Demographic Questionnaire, was categorized by below 130% of the federal poverty level for that year (ie, <30% above the poverty level) or at or above 130%, consistent with eligibility for governmental assistance including the Supplemental Nutrition Assistance Program.^18^

#### Race and Hispanic Ethnicity Categories

In NHANES, participants are given the option of self-identifying as Hispanic or Latino, including country of self or ancestral origin. Participants then self-identify race and are allowed to select multiple; if other race is selected, additional subcategories are presented, including Asian subcategories.^19^ The publicly available aggregated racial categories included non-Hispanic Asian, Black, and White, and more than 1 or other race (which could include American Indian or Alaska Native, Native Hawaiian or Other Pacific Islander, and other), while ethnicity categories included Mexican American or Other Hispanic.

### Outcomes: Adjusted Prediabetes Prevalence and Prevalence Ratios

Prediabetes was defined only by HbA_1c_ level due to the markedly smaller sample of adolescents with available fasting glucose levels and potential nonrepresentativeness of this small subpopulation. Using American Diabetes Association criteria,^20^ HbA_1c_ levels of 5.7% to 6.4% were considered consistent with prediabetes; 6.5% or greater, T2D (to convert to mmol/mol, multiply by 10.93 and subtract by 23.50).

### Potential Confounders

We adjusted for age, sex assigned at birth, and relative obesity (BMI *z* score). The BMI *z* score was calculated using US Centers for Disease Control and Prevention pediatric growth curves^21^ and the zanthro command in Stata, version 17 (StataCorp LLC).

### Statistical Analysis

Demographic and clinical characteristics are reported using summary statistics and corresponding 95% CIs. We estimated associations between each SDOH and prediabetes prevalence using multivariable logistic regression models, adjusted for confounders. We included interaction terms for race and ethnicity and each SDOH. White race was used as the reference group due to being the largest racial or ethnic group. We calculated point estimates and 95% CIs of adjusted prediabetes prevalence by presence or absence of each adverse SDOH category (eg, food insecurity vs food security [reference group]; any vs no adverse SDOH) within racial and ethnic groups.

Analyses were performed using Stata, version 17. All analyses incorporated appropriate sample weights^22^ and used a 2-sided α = .05 to indicate statistical significance. Due to repeated testing of the association between SDOH and prediabetes prevalence within multiple racial and ethnic groups (6 groups), false discovery rate–adjusted *P* values are reported. We used Stata survey procedures to ensure appropriate point and variance estimates for our study’s population. Missing data were not imputed. Relative SEs were calculated to evaluate reliability of estimates; estimates with a relative SE of 30% or greater (less reliable) are reported herein.^23^ Analyses were performed from June 1, 2023, to April 5, 2024.

## Results

### Cohort Characteristics

Of the 4522 participants aged 12 to 18 years old with NHANES data available from the 2011-2018 cycles, 1563 had a BMI at or above the 85th percentile for age and sex, an available HbA_1c_ level measurement, and a reported diabetes diagnosis status. The final analytic sample of 1563 participants representing 10 178 400 US youth had a mean age of 15.5 (95% CI, 15.3-15.6) years and BMI *z* score of 1.77 (95% CI, 1.74-1.80). The sample was evenly distributed between male and female participants (50.5% [95% CI, 47.1%-53.9%] female and 49.5% male [95% CI, 46.1%-52.9%]), and approximately half were of White race (weighted proportion: Asian, 3.0% [95% CI, 2.2%-3.9%]; Black, 14.9% [95% CI, 11.6%-19.1%]; Mexican American, 18.8% [95% CI, 15.4%-22.9%]; Other Hispanic, 8.1% [95% CI, 6.5%-10.1%]; White, 49.1% [95% CI, 43.2%-55.0%]; and >1 or other race, 6.1% [95% CI, 4.6%-8.0%]). Elevated HbA_1c_ level (≥5.7%) was present in 8.5% (95% CI, 6.9%-10.4%) but varied by race and ethnicity, with the largest proportion in Asian (14.3% [95% CI, 8.8%-22.4%]) and Black (24.5% [95% CI, 19.4%-30.7%]) youths, followed by Mexican American (9.7% [95% CI, 5.9%-15.8%]), Other Hispanic (7.9% [95% CI, 4.6%-13.3%]), more than 1 or other race (6.6% [95% CI, 2.9%-14.1%]), and White (3.1% [9% CI, 1.9%-4.9%]) youths. Diabetes-range HbA_1c_ levels were uncommon, occurring in only 3 individuals.

### Prevalence of Adverse SDOH

Food insecurity was reported by 41.0% (95% CI, 37.6%-45.5%) of youths overall (available in an unweighted sample of 1534 participants). More than half reported no private health insurance coverage (no insurance, 10.2% [95% CI, 8.4%-12.4%]; public insurance, 43.2% [95% CI, 39.4%-46.9%]; available in an unweighted sample of 1558 participants). Approximately one-third (35.6% [95% CI, 31.6%-39.8%]) reported low household income (income to poverty ratio <130%) (available in an unweighted sample of 1420 participants). When considered jointly (among an unweighted sample of 1416 participants with data for food security, insurance, and income), the experience of at least 1 adverse SDOH was pervasive, with 67.0% (95% CI, 63.0%-70.7%) reporting food insecurity, lack of private insurance, and/or low household income, and 20.5% (95% CI, 17.1%-24.3%) reporting all 3 adverse SDOH. The unadjusted prevalence of each investigated SDOH varied across racial and ethnic groups (*P* < .001 for each by χ^2^ test), but adverse SDOH, for example food insecurity, were generally most common among Black (55.7% [95% CI, 50.2%-61.1%]), Mexican American (55.5% [95% CI, 48.4%-62.3%]), and Other Hispanic (51.9% [95% CI, 40.7%-62.9%]) youths and lowest among Asian (26.2% [95% CI, 15.4%-40.8%]) and White (29.6% [95% CI, 23.8%-36.2%) youths (Table 1).

**Table 1.** Prevalence of Adverse SDOH by Race and Ethnicity

Adverse SDOH	Racial and ethnic groups, prevalence (95% CI), %^a^					
	Asian	Black	Mexican American	Other Hispanic	White	>1 or Other race^b^
Less than full food security	26.2 (15.4-40.8)	55.7 (50.2-61.1)	55.5 (48.4-62.3)	51.9 (40.7-62.9)	29.6 (23.8-36.2)	45.3 (33.5-57.7)
Nonprivate insurance						
Public	29.5 (19.7-41.7)	60.3 (55.7-64.8)	53.3 (47.1-59.3)	55.8 (47.2-64.1)	33.2 (28.7-38.0)	40.1 (30.3-50.8)
None	6.8 (2.8-15.6)^c^	8.6 (6.1-12.1)	18.7 (14.2-24.0)	15.1 (9.4-23.4)	6.7 (4.2-10.5)	11.8 (5.3-24.3)^c^
Household income <130% poverty level	29.4 (19.4-41.9)	49.3 (43.3-55.4)	51.5 (44.6-58.5)	51.0 (42.3-59.6)	24.3 (19.7-29.5)	33.5 (23.5-45.2)
Any adverse SDOH	52.4 (40.3-64.3)	85.9 (81.7-89.3)	83.4 (78.5-87.3)	82.7 (76.0-87.8)	54.0 (48.3-59.7)	67.4 (56.6-76.6)
Cumulative adverse SDOH						
1	21.1 (13.9-30.8)	29.0 (23.5-35.2)	21.7 (17.0-27.4)	23.0 (15.4-32.9)	26.6 (21.6-32.3)	24.2 (14.6-37.3)
2	21.0 (12.8-32.4)	24.8 (20.4-29.9)	31.0 (25.7-36.8)	27.7 (20.0-37.1)	15.2 (11.0-20.6)	21.8 (12.6-35.0)
3	10.3 (4.4-22.3)	32.0 (26.6-38.0)	30.7 (24.2-38.0)	32.0 (23.1-42.4)	12.2 (8.5-17.3)	21.4 (12.4-34.5)

Abbreviation: SDOH, social determinants of health.

^a^ Unweighted sample sizes with responses for each measure included 1534 for food security, 1544 for health insurance, 1520 for income, and 1416 youths for any and cumulative SDOH. Data are from the 2011 to 2018 cycles of the National Health and Nutrition Examination Survey including youths aged 12 to 18 years with a body mass index at or above the 85th percentile without known diabetes. *P* < .001 for all between-group differences.

^b^ Other includes American Indian or Alaska Native, Native Hawaiian or Other Pacific Islander, and other race.

^c^ Estimate may be less reliable owing to a relative SE of 30% or greater.

### Prevalence of Prediabetes by Adverse SDOH and by Intersectional Racial and Ethnic SDOH Group

Adjusted marginal prevalence of prediabetes by SDOH groups are shown in the Figure. Prediabetes prevalence was 4.1% (95% CI, 0.7%-7.5%) higher among youths from households with food insecurity compared with food security. Similarly, prediabetes prevalence was 5.3% (95% CI, 1.6%-9.1%) higher among youths with public compared with private insurance, though prevalence did not differ significantly between noninsured and privately insured youths (4.6% [95% CI, −1.2% to 10.3%]). Prediabetes prevalence was also 5.7% (95% CI, 3.0%-8.3%) higher among youths with household income at less than 130% of the federal poverty level compared with at least 130%.

**Figure.**
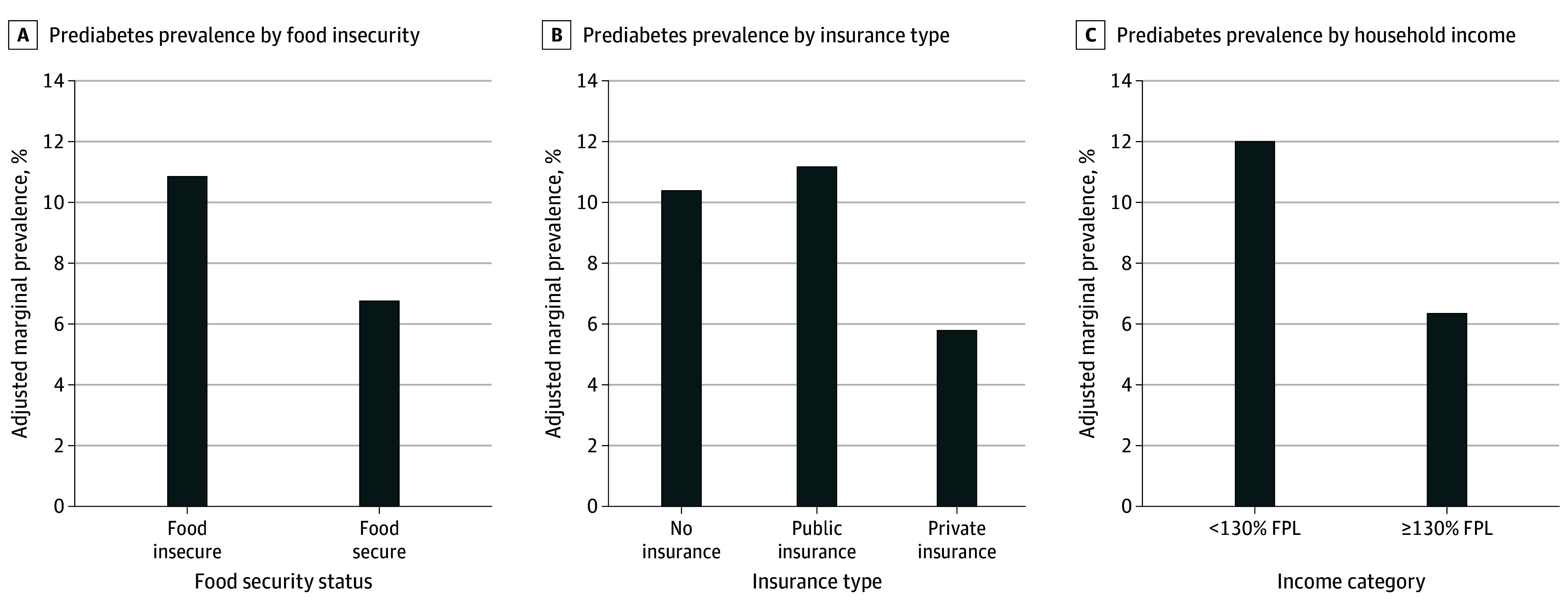
Adjusted Marginal Prediabetes Prevalence Among Youth With Adverse Social Determinants of Health Prediabetes prevalence was higher among youth with food insecurity, public health insurance, and income to poverty ratio less than 130% of the federal poverty level (FPL) (*P* < .05 for each comparison).

Adjusted marginal prevalences of prediabetes by individual SDOH within racial and ethnic groups are shown in Table 2. Food insecurity (marginal, low, and very low) was associated with significantly higher prediabetes prevalence only among White youths (6.3% [95% CI, 2.8%-9.7%]) (Table 2). Use of public vs private insurance was associated with significantly higher prediabetes prevalence among Asian (32.0% [95% CI, 15.3%-48.6%]) and White (7.1% [95% CI, (3.4%-10.9%]) youths (Table 2). Low income was associated with significantly higher prediabetes prevalence among Black (28.5% [95% CI, 20.4%-36.5%]) and White (6.3% [95% CI, 3.5%-9.2%]) youths (Table 2). When the additive effect of each of the 3 SDOH was evaluated, although point estimates suggested a gradient of risk difference across several groups, prediabetes prevalence was significantly higher only for White youths with 2 (8.1% [95% CI, 1.6%-15.1%]) or 3 (8.3% [95% CI, 4.9%-11.8%]) compared with 0 (0.6% [95% CI, −0.7% to 2.0%]) adverse SDOH (Table 2).

**Table 2.** Prediabetes Prevalence by Intersectional Adverse SDOH Status and Racial and Ethnic Identity

SDOH category	Racial or ethnic group^a^											
	Asian		Black		Mexican American		Other Hispanic		White		>1 or Other race^b^	
	Prevalence (95% CI), %	*P* value^c^	Prevalence (95% CI), %	*P* value^c^	Prevalence (95% CI), %	*P* value^c^	Prevalence (95% CI), %	*P* value^c^	Prevalence (95% CI), %	*P* value^c^	Prevalence (95% CI), %	*P* value^c^
Food security status												
Marginal, low, or very low	27.3 (9.6 to 44.9)	.20	23.9 (18.0 to 29.8)	.55	10.5 (3.5 to 17.5)	.50	8.8 (2.2 to 15.5)	.80	6.3 (2.8 to 9.7)	.02	9.4 (0.4 to 18.4)	.20
Secure (reference)	12.2 (3.1 to 21.3)	NA	21.0 (13.1 to 29.0)	NA	7.6 (3.2 to 12.0)	NA	7.7 (2.3 to 13.2)	NA	1.6 (0.2 to 3.0)	NA	2.8 (−1.0 to 6.7)	NA
Insurance												
None	8.6 (−6.9 to 24.0)^d^	>.99	20.5 (6.3 to 34.8)	>.99	9.1 (1.0 to 17.2)	.50	5.0 (−0.9 to 10.8)	.07	6.6 (−3.0 to 16.2)	.20	5.9 (−6.7 to 18.5)^d^	.80
Public	32 (15.3 to 48.6)	.01	23.8 (17.8 to 29.8)	.60	10.5 (3.8 to 17.2)	.20	6.5 (0.5 to 12.5)	.70	7.1 (3.4 to 10.9)	.002	7.9 (−0.2 to 16.1)	.40
Private (reference)	8.6 (1.5 to 15.7)	NA	20.8 (12.0 to 29.6)	NA	5.9 (1.3 to 10.4)	NA	19.6 (4.7 to 34.5)	NA	0.8 (−0.3 to 1.8)	NA	4.3 (−0.5 to 9.0)	NA
Ratio of income to FPL												
<130%	26.0 (8.7 to 43.4)	.10	28.5 (20.4 to 36.5)	.02	11.3 (4.9 to 17.8)	.40	12.1 (4.5 to 19.7)	.20	6.3 (3.5 to 9.2)	.01	12.6 (−0.6 to 25.7)	.10
≥130% (Reference)	11.5 (3.9 to 19.2)	NA	18.2 (12.3 to 24.0)	NA	8.1 (2.7 to 13.5)	NA	5.3 (0.1 to 10.5)	NA	2.3 (0.5 to 4.0)	NA	2.8 (−0.4 to 6.0)	NA
No. of adverse SDOH												
3	38.2 (9.2 to 67.1)	.08	31.3 (22.0 to 40.6)	.70	12.2 (2.9 to 21.6)	.20	9.8 (1.1 to 18.4)	.20	8.3 (4.9 to 11.8)	<.001	16.6 (−1.3 to 34.4)	.20
2	22.8 (2.9 to 42.6)	.40	19.6 (10.6 to 28.5)	.20	9.5 (2.1 to 16.9)	.40	13.5 (3.2 to 23.9)	.06	8.3 (1.6 to 15.1)	.03	1.3 (−1.4 to 3.9)	.40
1	5.6 (−3.1 to 14.4)	.10	16.1 (9.0 to 23.1)	.10	9 (2.1 to 16.0)	.30	6.4 (−2.2 to 15.1)	.40	1.9 (−0.5 to 4.3)	.40	2.3 (−2.3 to 7.0)	.70
0 (Reference)	12.1 (2.4 to 21.8)	NA	27.9 (14.1 to 41.6)	NA	5.5 (−0.5 to 11.4)	NA	2.5 (−2.7 to 7.6)	NA	0.6 (−0.7 to 2.0)	NA	4.0 (−1.7 to 9.6)	NA

Abbreviations: FPL, federal poverty level; NA, not applicable; SDOH, social determinants of health.

^a^ Unweighted sample sizes with responses for each measure included 1534 for food security, 1558 for health insurance, 1420 for income, and 1416 youths for cumulative SDOH. Multivariable logistic regression models were adjusted for age, sex, body mass index *z* score, and survey cycle. Data are from the 2011 to 2018 cycles of the National Health and Nutrition Examination Survey including youths aged 12 to 18 years with a body mass index at or above the 85th percentile without known diabetes.

^b^ Other includes American Indian or Alaska Native, Native Hawaiian or Other Pacific Islander, and other race.

^c^ False discovery rate–adjusted *P* value for comparisons within each of 6 racial and ethnic categories; reference category is the most advantaged group.

^d^ Estimate may be less reliable owing to relative SE of 30% or greater.

## Discussion

In this cross-sectional study including a nationally representative sample of adolescents with overweight and obesity, prediabetes prevalence was higher in the presence of adverse SDOH for the full subsample, but differing associations were found across racial and ethnic groups. To our knowledge, our study is the first to evaluate prediabetes prevalence in youth using intersectional race, ethnicity, and SDOH categories. This approach allowed us to isolate the association between prediabetes and each adverse SDOH while also highlighting how the association may differ by racial or ethnic identity. Our findings are similar to those from a cross-sectional study by Ogden et al^24^ that demonstrated differing associations between obesity and household income among youth across race and ethnicity groups, with high household income associated with lower obesity prevalence among Asian and Hispanic youths, but no income-related difference among White and Black youths. Similarly, a scoping review of associations between SDOH and health risk behaviors (eg, substance use, high-risk sexual behavior) among adolescents and young adults aged 10 to 24 years^25^ found that associations differed across racial and ethnic groups and by SDOH assessed. These findings underscore the complexity of the associations among racial and ethnic identity, exposure to adverse SDOH, and health outcomes in youths.

Differences in T2D risk across and within racial and ethnic groups^26^ emerge from factors at the individual, interpersonal, community, and societal levels.^27^ Youth-onset prediabetes results from a complex interplay across these levels, on an intergenerational scale. For example, risk of youth-onset obesity and insulin resistance is significantly higher among offspring of women with diabetes during pregnancy,^9,28^ but this maternal risk differs by race and ethnicity^29^ and is driven by exposure to adverse SDOH.^30,31^ Youth-onset obesity and prediabetes risk are reduced by engagement in health-promoting behaviors, including physical activity and consumption of nutrient-dense foods; unfortunately, these health behaviors are often hardest to achieve for youths from communities which, due to oppressive policies and practices, have concentrated poverty and high rates of community violence.^9^ We also note that the prevalence of adverse SDOH in this cohort was relatively higher than the general population. Thus, our analytic approach of evaluating within-racial and ethnic group differences minimizes the confounding of associations between SDOH and prediabetes risk that occur due to differences in adverse SDOH exposure across racial and ethnic groups.

Our study also highlights differences in prediabetes prevalence among Asian youths, an understudied population at high risk for T2D.^32,33^ Asian American individuals are a heterogenous group, with vast differences in socioeconomic status, language identity, and immigration status both between and within cultural subgroups.^34^ Unfortunately, unrestricted NHANES data do not allow for disaggregation of this heterogenous group, an important limitation given the differing effects of SDOH within Asian subgroups. For example, educational attainment was found to be inversely associated with T2D risk among Filipino American adults, but directly associated with T2D risk among Indian American adults.^35^ Within Asian adults, those of South Asian extraction tend to be at highest risk, with T2D developing approximately 10 years earlier than among White adults.^36^ Adverse SDOH further exacerbate this risk: low household income is associated with higher prevalence of obesity among Asian American adolescents^37^ as well as with prediabetes and T2D among Asian American adults.^38^ Disaggregating data could allow for better identification of drivers of health disparities within Asian and Pacific Islander communities, supporting the development of effective diabetes prevention interventions.

### Strengths and Limitations

A major strength of our study is our evaluation of associations within racial and ethnic groups between adverse SDOH and prediabetes prevalence, which provided a less confounded estimate of associations. We observed consistently direct associations between adverse SDOH and prediabetes prevalence through analyses of individual, any, and cumulative adverse SDOH. Additional strengths include the nationally representative sample of youths with rigorously and prospectively collected clinical and demographic data, including self-reported racial and ethnic identity.

This study also has some limitations. First, we did not include alternate, glucose-based prediabetes definitions. Although HbA_1c_ level has been reported to be higher among certain racial groups,^39^ we defined prediabetes using HbA_1c_ because (1) an elevated HbA_1c_ level has been associated with development of T2D in diverse populations, including American Indian youth^40^; (2) it is guideline supported^5^ and commonly used to screen for, diagnose, and manage T2D in youths^41,42^; (3) it is not influenced by fasting and reflects longer-term glycemia^20^; and, (4) our within-group analysis minimizes concerns for between-race differences in glycosylation. Second, detailed family history was not available, so we could not adjust for this potential confounder. Third, we did not evaluate whether prevalence differences were associated with nutrition or physical activity. However, in a study also using NHANES data,^43^ diet quality was lowest among Black youths, independent of income, suggesting that diet may be an important factor explaining some of the observed racial differences in prediabetes prevalence. Fourth, NHANES aggregates individuals of other race and more than 1 race, an important limitation given the heterogeneity and increasing size of this population in the US.^44^ Fifth, adverse SDOH may be differentially reported by adolescents and caregivers, potentially leading to underestimation^45^ or overestimation^46^ of material needs. Last, this cross-sectional study does not allow us to determine causal relationships between SDOH and prediabetes.

## Conclusions

In this cross-sectional study including a nationally representative sample of 1563 adolescents with overweight and obesity aged 12 to 18 years, we found that adverse SDOH, including food insecurity, lack of private health insurance, and low income were differentially associated with prediabetes prevalence across racial and ethnic groups. However, prediabetes prevalence remained high even in the setting of favorable SDOH among Asian, Black, and Hispanic youth. Other social and structural determinants of health, such as racism and ethnic discrimination, should be evaluated to determine whether the remaining disparities in prediabetes prevalence can be further explained to develop targeted approaches to T2D risk reduction. Furthermore, given the higher prevalence of prediabetes in the setting of SDOH, even in White youth who are not considered to be at high risk for T2D,^5^ pediatric T2D screening guidelines should move beyond use of race and ethnicity and instead critically consider exposure to adverse SDOH.^47^ Such an approach would be well aligned with recent efforts by many pediatric health care organizations to make screening for health-related social needs standard of care.^48^ If successfully implemented, approaches that screen for and address adverse SDOH may ultimately reduce the risks associated with youth-onset T2D via prevention as well as early identification and treatment.

## Notes

Supplement 1. **eTable.** Questions and Questionnaires Used, Target Age Range, and Respondent Detail **eFigure.** Consort Diagram

Supplement 2.
Data Sharing Statement
