# Supplementary material for: Prediabetes Prevalence by Adverse Social Determinants of Health in Adolescents
Source: JAMA Netw Open. 2024 Jun 11;7(6):e2416088. doi: 10.1001/jamanetworkopen.2024.16088 (PMC11167496; doi:10.1001/jamanetworkopen.2024.16088)
Supplement: Supplement 1. — eTable. Questions and Questionnaires Used, Target Age Range, and Respondent Detail eFigure. Consort Diagram [file jamanetwopen-e2416088-s001.pdf]

Supplementary Online Content

Harrison C, Peyyety V, Rodriguez Gonzalez A, et al. Prediabetes prevalence by adverse social determinants of health in adolescents. *JAMA Netw Open*. 2024;7(6):e2416088. doi:10.1001/jamanetworkopen.2024.16088

eTable. Questions and Questionnaires Used, Target Age Range, and Respondent Detail

eFigure. Consort Diagram

This supplementary material has been provided by the authors to give readers additional information about their work.

© 2024 Harrison C et al. *JAMA Network Open*.

**eTable.** Questions and Questionnaires Used, Target Age Range, and Respondent Detail					
**Question number**	**Questionnaire**	**Cycle**	**Topic**	**Age range**	**Respondent (Child = C, Parent = P)**
DIQ010	DIQ_J	2011-2018	Doctor told you have diabetes	0-150 years	C≥16y, P<16y
DIQ160	DIQ_J	2011-2018	Ever told you have prediabetes	12-150 years	C≥16y, P<16y
FSDHH	FSQ	2011-2018	Household food security category	0-150 years	P
INDFMPIR	DEMO	2011-2018	Ratio of family income to poverty	0-150 years	P
HIQ011	HIQ	2011-2018	Covered by health insurance	0-150 years	C≥16y, P<16y
HIQ031a	HIQ	2011-2018	Covered by private insurance	0-150 years	C≥16y, P<16y
HIQ031b	HIQ	2011-2018	Covered by Medicare	0-150 years	C≥16y, P<16y
HIQ031c	HIQ	2011-2018	Covered by Medi-Gap	0-150 years	C≥16y, P<16y
HIQ031d	HIQ	2011-2018	Covered by Medicaid	0-150 years	C≥16y, P<16y
HIQ031e	HIQ	2011-2018	Covered by CHIP	0-150 years	C≥16y, P<16y
HIQ031f	HIQ	2011-2018	Covered by military health care	0-150 years	C≥16y, P<16y
HIQ031h	HIQ	2011-2018	Covered by state-sponsored health plan	0-150 years	C≥16y, P<16y
HIQ031i	HIQ	2011-2018	Covered by other government insurance	0-150 years	C≥16y, P<16y
HIQ031j	HIQ	2011-2018	Covered by single service plan	0-150 years	C≥16y, P<16y
HIQ031aa	HIQ	2011-2018	No coverage of any type	0-150 years	C≥16y, P<16y

© 2024 Harrison C et al. *JAMA Network Open*.

eFigure. Consort Diagram

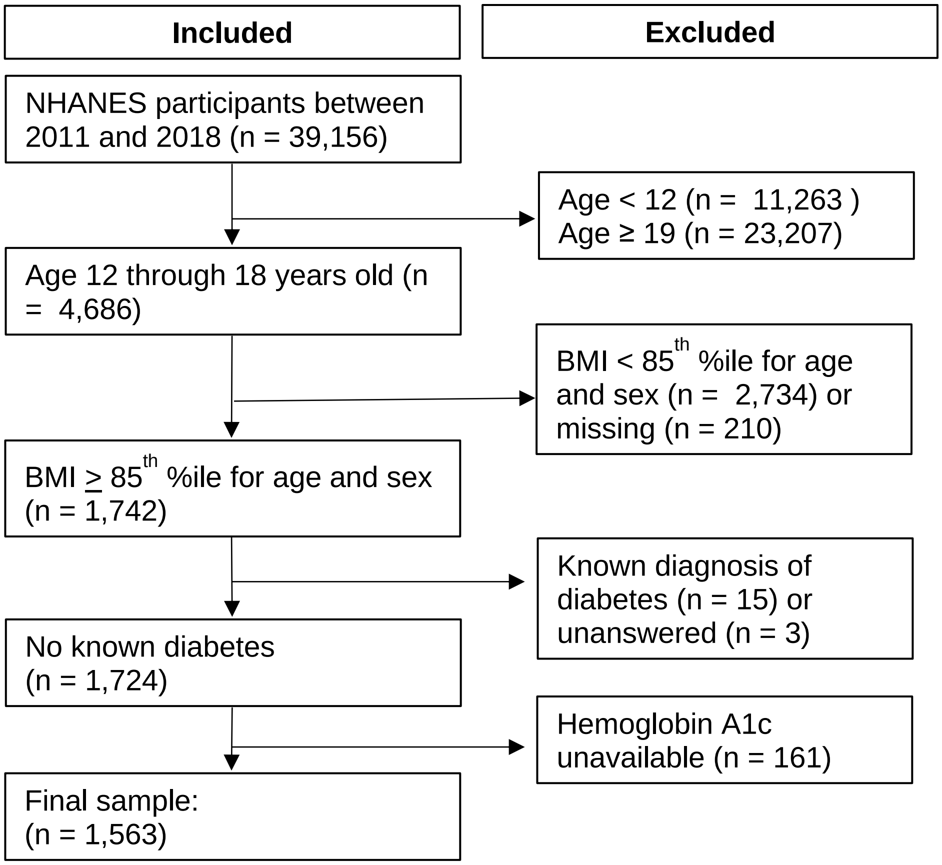

© 2024 Harrison C et al. *JAMA Network Open*.
