# Supplementary material for: Prediabetes Prevalence by Adverse Social Determinants of Health in Adolescents
Source: JAMA Netw Open. 2024 Jun 11;7(6):e2416088. doi: 10.1001/jamanetworkopen.2024.16088 (PMC11167496; doi:10.1001/jamanetworkopen.2024.16088)
Supplement: Supplement 2. — Data Sharing Statement [file jamanetwopen-e2416088-s002.pdf]

Data Sharing Statement

Harrison. Prediabetes Prevalence by Adverse Social Determinants of Health in Adolescents. *JAMA Netw Open*. Published June 11, 2024. doi:10.1001/jamanetworkopen.2024.16088

Data
Data available: No

Additional Information
Explanation for why data not available: NHANES dataset is publicly available
